# The NLR Protein Encoded by the Resistance Gene *Ty-2* Is Triggered by the Replication-Associated Protein Rep/C1 of Tomato Yellow Leaf Curl Virus

**DOI:** 10.3389/fpls.2020.545306

**Published:** 2020-09-10

**Authors:** Xuexue Shen, Zhe Yan, Xiaoxuan Wang, Yinlei Wang, Marjon Arens, Yongchen Du, Richard G. F. Visser, Richard Kormelink, Yuling Bai, Anne-Marie A. Wolters

**Affiliations:** ^1^ Plant Breeding, Wageningen University & Research, Wageningen, Netherlands; ^2^ Graduate School Experimental Plant Sciences, Wageningen University & Research, Wageningen, Netherlands; ^3^ Institute of Vegetable and Flowers, Chinese Academy of Agricultural Sciences, Beijing, China; ^4^ Institute of Vegetable Crops, Jiangsu Academy of Agricultural Sciences, Nanjing, China; ^5^ Jiangsu Key Laboratory for Horticultural Crop Genetic Improvement, Nanjing, China; ^6^ Laboratory of Virology, Wageningen University & Research, Wageningen, Netherlands

**Keywords:** TYLCV, *Ty-2*, cloning, NLR gene, Rep/C1, avirulence factor

## Abstract

The whitefly-transmitted tomato yellow leaf curl virus (TYLCV) is one of the most destructive viral pathogens of cultivated tomato. To combat TYLCV, resistance gene *Ty-2* has been introduced into cultivated tomato (*Solanum lycopersicum*) from wild tomato species *Solanum habrochaites* by interspecific crossing. Introgression lines with *Ty-2* contain a large inversion compared with *S. lycopersicum*, which causes severe suppression of recombination and has hampered the cloning of *Ty-2* so far. Here, we report the fine-mapping and cloning of *Ty-2* using crosses between a *Ty-2* introgression line and several susceptible *S. habrochaites* accessions. *Ty-2* was shown to encode a nucleotide-binding leucine-rich repeat (NLR) protein. For breeding purposes, a highly specific DNA marker tightly linked to the *Ty-2* gene was developed permitting marker-assisted selection. The resistance mediated by *Ty-2* was effective against the Israel strain of TYLCV (TYLCV-IL) and tomato yellow leaf curl virus-[China : Shanghai2] (TYLCV-[CN : SH2]), but not against tomato yellow leaf curl Sardinia virus (TYLCSV) and leafhopper-transmitted beet curly top virus (BCTV). By co-infiltration experiments we showed that transient expression of the Rep/C1 protein of TYLCV, but not of TYLCSV triggered a hypersensitive response (HR) in *Nicotiana benthamiana* plants co-expressing the *Ty-2* gene. Our results indicate that the *Rep*/*C1* gene of TYLCV-IL presents the avirulence determinant of *Ty-2-*mediated resistance.

## Introduction

Tomato yellow leaf curl disease (TYLCD) is a viral disease caused by a complex of virus species. Tomato yellow leaf curl virus (TYLCV) is one of the causing agents and the most wide-spread one (Navas-Castillo et al., 2011). TYLCV belongs to the *Begomovirus* genus in the *Geminiviridae* family (Jeske, 2018). Its genome consists of one circular single-stranded DNA molecule containing six open reading frames (ORFs) (Gronenborn, 2007). Based on their functions, the proteins encoded by the six ORFs have been named coat protein (CP/V1), virus movement protein (MP/V2), replication-associated protein (Rep/C1), transcriptional activation protein (TrAP/C2), replication enhancer protein (REn/C3), and a protein determining symptom expression and virus spreading (C4). All six proteins are essential for successful systemic infection of host plants (Castillo et al., 2007). Besides tomato (*Solanum lycopersicum* L.), TYLCV has been detected in various economically important crops and weed species including sweet pepper (*Capsicum annuum* L.), chili pepper (*Capsicum chinense* Jacq.), tobacco (*Nicotiana tabacum* L.), common bean (*Phaseolus vulgaris* L.), petunia (*Petunia × hybrida* hort. ex E. Vilm.), and lisianthus [*Eustoma grandiflorum* (Raf.) Shinners] (Díaz-Pendón et al., 2010). Under natural conditions, TYLCV is transmitted exclusively by whiteflies (*Bemisia tabaci* Genn.). In practice, preventing viruses from infecting the host mainly requires the control of virus vectors by the use of appropriate physical barriers (traps and screens) and chemical agents (insecticides). However, building effective physical barriers is not always feasible and the application of chemical compounds can result in the development of resistance against the used compound by whiteflies (Roditakis et al., 2009; Luo et al., 2010). Because of the broad host range and the difficulties to control the vector, TYLCD has a serious economic impact on the production of agricultural crops. To minimize the damage caused by TYLCV and to avoid excessive use of insecticides, the most important crop protection method is breeding crops with resistance against whiteflies and/or viruses. In tomato breeding for TYLCV resistance, the most prominent approach is transferring virus resistance genes from wild tomato relatives into cultivated tomato. To date, six resistance genes (*Ty-1*, *Ty-2*, *Ty-3*, *Ty-4*, *ty-5*, and *Ty-6*) identified from several wild tomato species have been mapped on tomato chromosomes (Zamir et al., 1994; Hanson et al., 2000; Ji et al., 2007; Anbinder et al., 2009; Ji et al., 2009b; Hutton and Scott, 2014). So far, *Ty-1*, *Ty-2*, and *Ty-3* are the main sources of resistance for tomato breeding programs. *Ty-1*, *Ty-3*, and *ty-5* have been cloned (Verlaan et al., 2013; Lapidot et al., 2015) and involve two different antiviral mechanisms. *Ty-1* and *Ty-3* are alleles of the same gene encoding an RNA-dependent RNA polymerase (RDR) involved in transcriptional gene silencing (TGS). An increased production of viral small RNAs (sRNAs) and an enhanced cytosine methylation of the viral DNA was detected in *Ty-1* tomato plants challenged with TYLCV (Butterbach et al., 2014). The gene confers resistance not only to strains of TYLCV and other *Begomoviruses*, but also to the leafhopper-transmitted Beet curly top *Curtovirus* (BCTV) (Voorburg et al., 2020). The *ty-5* gene encodes messenger RNA (mRNA) surveillance factor *Pelota*. Loss-of-function allele *ty-5* hinders viral translation, leading to resistance (Lapidot et al., 2015), indicating that the *Pelota* gene is a susceptibility gene for TYLCV.

The completely dominant *Ty-2* gene has been reported in the tomato line H24 (Kalloo and Banerjee, 1990) derived from *Solanum habrochaites* S. Knapp & D.M. Spooner accession B6013 (Banerjee and Kalloo, 1987). The gene confers a high level of resistance to the Israel strain of TYLCV (TYLCV-IL) (Chen et al., 2013; Shahid et al., 2013), but not to several other TYLCV-like viruses. When infected with tomato yellow leaf curl Sardinia virus (TYLCSV) (Barbieri et al., 2010) and an isolate of the Mild strain of TYLCV (TYLCV-Mld) (Ohnishi et al., 2016), *Ty-2-*carrying plants exhibit systemic infection. An artificial chimeric strain containing the genomic region with overlapping *Rep*/*C1* and *C4* genes from the TYLCV-Mld strain in the TYLCV-IL background induces systemic infection in *Ty-2-*containing plants, pointing toward their role as putative avirulence determinant of *Ty-2-*mediated resistance (Ohnishi et al., 2016).

*Ty-2* was first mapped on the long arm of chromosome 11 by Hanson et al. (2000). Our continuing efforts to clone the *Ty-2* gene has proven to be a time-consuming process due to the suppression of recombination in the *Ty-2* genomic region (Yang et al., 2014), where a chromosomal inversion is present in *S. habrochaites* compared with *S. lycopersicum* (Wolters et al., 2015). To circumvent the barrier of suppressed recombination between the *Ty-2* introgression line and cultivated tomato, we started to utilize crosses in which the genomic region harboring *Ty-2* was always from a *S. habrochaites* origin. To this end, crosses were made between the *Ty-2* introgression line and several TYLCV-susceptible *S. habrochaites* accessions (Wolters et al., 2015). Using this strategy, multiple recombinants have been obtained that ultimately facilitated fine-mapping and cloning of *Ty-2.* Meanwhile, Yamaguchi and co-workers (2018) sequenced the upper and lower ends of the inversion in the *Ty-2* region of resistant and susceptible lines, which allowed them to identify two candidate nucleotide-binding leucine-rich repeat (NLR) genes of which one was eventually shown to be the *Ty-2* gene by functional complementation.

To date, more than 200 antiviral NLR genes have been reported from different plant species. From those about 28 have been cloned that act against plant viruses of which only two confer resistance to *Begomoviruses* (de Ronde et al., 2014a; Boualem et al., 2016; Yamaguchi et al., 2018). *PvVTT1* confers resistance to bean dwarf mosaic virus (BDMV) and *CYR1* to mung bean yellow mosaic India virus (MYMIV) (Seo et al., 2007; Maiti et al., 2012). The resistance conferred by *PvVTT1* and *CYR1* involve recognition of the nuclear shuttle protein (NSP) of BDMV and the coat protein (CP) of MYMIV, respectively (Garrido-Ramirez et al., 2000; Zhou et al., 2007; Maiti et al., 2012).

The present study aimed to identify the TYLCV viral protein triggering tomato resistance gene *Ty-2*. Prior to this, the first part of this study describes our efforts on overcoming the difficulties in cloning of the *Ty-2* gene caused by chromosomal inversion *via* a map-based cloning approach. During this process a highly specific DNA marker tightly linked to the *Ty-2* gene was developed. Subsequently, we show that transient expression of the TYLCV *Rep*/*C1* gene, but not the *C4* gene, consistently triggered a hypersensitive response (HR) in *Nicotiana benthamiana* plants co-expressing the *Ty-2* gene. Using virus species/strains that differ in their ability to overcome the *Ty-2* resistance, the amino acid changes in the Rep/C1 protein were analyzed. Our results indicate that the Rep/C1 protein of TYLCV presents the avirulence determinant of the *Ty-2* gene.

## Materials And Methods

### Plant Materials

A *Ty-2* introgression line (hereafter named the *Ty-2* line) derived from H24 (Kalloo and Banerjee, 1990) was obtained from a breeding company. This line was selfed to produce PV123173. *S. lycopersicum* ‘Moneymaker’ (MM), PV123173, and its selfed progeny, and progeny from crosses between these genotypes were available or produced at WUR-plant breeding (Yang et al., 2014; Wolters et al., 2015). TYLCV-susceptible *S. habrochaites* accessions G1.1560 (CGN15790), G1.1257 (CGN15370), G1.1290 (CGN15391) were obtained from the Centre for Genetic Resources (CGN), Wageningen, the Netherlands. F_1_ families were developed between the *Ty-2* line and the three susceptible *S. habrochaites* accessions. F_1_ plants were backcrossed to the *Ty-2* line to obtain 10 BC_1_ families (PV155041, PV155044-52). An F_1_ plant from the cross between the *Ty-2* line and *S. habrochaites* accession G1.1257, which did not produce F_2_ seeds after selfing, was pollinated with bulked pollen from several F_1_ plants derived from the same cross. This resulted in pseudo-F_2_ family PV155053. Other F_1_ plants from all three susceptible *S. habrochaites* accessions could be selfed and produced F_2_ families. By selfing individual BC_1_ plants BC_1_S_1_ families were obtained. *S. lycopersicum, S. habrochaites*, derived progeny, and *Nicotiana benthamiana* plants were grown under greenhouse conditions (24°C with a 16 h light/8 h dark regime).

### *Agrobacterium*-Mediated Virus Inoculation

Disease assays were performed using *Agrobacterium*-mediated inoculation as described by Verlaan et al. (2011). The infectious clone of tomato yellow leaf curl virus-Israel strain TYLCV-IL (pTYCz40a, kindly provided by Dr. Eduardo Rodríguez Bejarano, Universidad de Malaga, Spain) was maintained in *Agrobacterium tumefaciens* strain LBA4404. The infectious clone of pBinPLUS-SH2-1.4A containing the full length genome (AM282874) of tomato yellow leaf curl virus-[China : Shanghai2] (TYLCV-[CN:SH2]) (kindly provided by Prof. Bingyan Xie, Chinese Academy of Agricultural Sciences, Beijing, China) was transformed to *A. tumefaciens* strain EHA105 as described by Zhang et al. (2009). The tomato yellow leaf curl Sardinia virus (TYLCSV) clone was provided by Dr. Emmanuela Noris [Institute for Sustainable Plant Protection (IPSP), Torino, Italy]. The beet curly top virus (BCTV) clone was kindly provided by Dr. Keith Saunders (John Innes Centre, Norwich, UK). *Agrobacterium* cultures containing the TYLCV-IL, TYLCV-[CN:SH2], or BCTV clone were used at an OD600 = 0.5 in the inoculations. For TYLCSV a higher concentration (OD600 = 1) was used. Agro-inoculation was performed on 3-week-old seedlings by pressure inoculation using a needleless syringe (Verlaan et al., 2011). To evaluate the spectrum of disease resistance, five plants of the *Ty-2* introgression line were agroinoculated per virus. Cultivar Moneymaker was included as susceptible control.

### Disease Assessment

TYLCV symptoms were visually evaluated using a 0 to 4 disease severity index (DSI) described by Friedmann et al. (1998), where 0 indicates no TYLCD symptoms, and 4 means severe TYLCD symptoms, including marked yellowing and curling of leaves and significant stunting of plants. Intermediate scores, 0.5, 1.5, 2.5, and 3.5 were incorporated for more precise disease severity scoring. For recombinant screening, plants having a DSI ≤ 1 were considered to be resistant, whereas plants with DSI >1 were rated as susceptible. To evaluate the resistance spectrum of the *Ty-2* gene, the average DSI and standard deviation of five tested plants was calculated. Relative virus accumulation was determined by qPCR. At 45–55 days after TYLCV or TYLCSV inoculation, top young leaves were harvested for DNA isolation using cetyltrimethyl ammonium bromide (CTAB) based protocol (Fulton et al., 1995). Viral accumulation was detected as described by Powell et al. (2012) using primers TYLCV-IS 1678F (TTCGTCTAGATATTCCCTATATGAGGAGGTA) and TYLCV-CONS 1756R (GGCAAGCCCATTCAAATTAAAGG) for TYLCV, and primers Q-TYLCSV-F1 (ATGCTACGGTTGTTGGAGGT) and Q-TYLCSV-R1 (TCGCCTGCTCTTGATGATTA) for TYLCSV. Tomato elongation factor 1α (*EF1α*) gene primers EF-F (ATTGGAAACGGATATGCCCCT) and EF-R (TCCTTACCTGAACGCCTGTCA) were used as reference. Relative fold-change of the ratio between viral and tomato DNA was calculated by the 2^−ΔΔCT^ method (Livak and Schmittgen, 2001). Values were normalized relative to tomato *EF1α* and referenced to the levels in MM plants (set to 1).

### Molecular Markers Used for Recombinant Screening

All markers used were PCR-based, including cleaved amplified polymorphic sequence (CAPS) markers, sequence characterized amplified region (SCAR) marker, and sequencing-based markers (**Supplementary Table S1**). These were either publicly available (Ji et al., 2009a; Yang et al., 2014) or were designed from newly obtained *Ty-2* line sequences by Primer 3 Plus online tool or OLIGO 6. CAPS markers used previously for *Ty-2* fine-mapping (Ji et al., 2009a; Yang et al., 2014) were tested for polymorphisms between three *S. habrochaites* accessions and the *Ty-2* introgression line. When possible, bi-allelic CAPS markers were developed that could be used to distinguish parental alleles. For all the markers, polymorphisms were detected between accession G1.1560 and the *Ty-2* line. Accessions G1.1257 and G1.1290 showed a relatively low level of polymorphism compared with the *Ty-2* line. For a few markers, PCR products were sequenced to identify differences between the parental alleles.

### Construction and Screening of Bacterial Artificial Chromosome Libraries

Two pooled bacterial artificial chromosome (BAC) libraries were screened to identify individual BACs containing sequences from the *Ty-2* introgression region. For both libraries *Hin*dIII fragments of *Ty-2*-containing lines were cloned. The first library with a 10x genome coverage was developed in China as described by Wolters et al. (2015). The second BAC library was developed by Bio S&T Inc. (St-Laurent, Quebec, Canada). DNA fragments of *Ty-2* line PV123173 (≥ 35x genome coverage) were cloned in pIndigoBAC (*Hin*dIII) vector and transformed to *Escherichia coli* DH10B cells. The average insert size was 140-kb. Colonies were distributed into six 96-deep well plates (pooled BAC library). Each well contained about 400 independent primary clones. A first screening of this BAC library was performed at Bio S&T Inc. by PCR using primer combinations AW52090F2R1 and AW9770F1R1 (**Supplementary Table S1**). Subsequent screenings were performed at WUR-plant breeding by PCR using primer pair AW910upF2R3 (**Supplementary Table S1**). Subpools were derived from positive pools and these were again tested by PCR. This was repeated until single positive BAC clones could be isolated.

### DNA Sequencing and Bioinformatics Analysis

Individual BAC clones were sequenced at INRA-CNRGV (Toulouse, France) on a PacBio RS sequencer using PacBio RS libraries with 8–12-kb insert size. A coverage of approximately 60x read depth per BAC was obtained. Sequences were aligned to *S. lycopersicum* Heinz genome SL2.50, to the available *S. habrochaites* accession LYC4 superscaffold described by Wolters et al. (2015) and to the *Solanum pennellii* Correll LA0276 genome version 1.0 by BLASTN analyses at National Center for Biotechnology Information (NCBI) and SGN (Sol Genomics Network) webpages and DNASTAR Lasergene 13 programs. BACs in the fine-mapped region were not overlapping. Therefore, gaps between the BAC sequences were filled by (long-range) PCR using primers from BAC ends, and Phusion High-Fidelity DNA polymerase (Thermo Scientific) or genome walking using the Universal GenomeWalker kit from Clontech. New primers were developed for sequencing the complete length of the PCR products. Sequences of PCR products were obtained by Sanger sequencing performed at GATC Biotech. The full-length sequence of the fine-mapped *Ty-2* region was compared with the *S. lycopersicum* Heinz genome sequence (versions SL2.50 and SL3.0) to identify possible candidate genes. Gene prediction in the fine-mapped *Ty-2* region was performed using FGENESH from SoftBerry (Solovyev et al., 2006) and Augustus (http://bioinf.uni-greifswald.de/augustus/).

### Cloning of *Ty-2* and *Ty-1* Genes and Virus-Encoded Proteins Into Overexpression Constructs

To clone the full-length complementary DNA (cDNA) of the *Ty-2* gene, the Maxima H Minus First Strand cDNA Synthesis Kit (Thermo Scientific) was used to synthesize cDNA using RNA of the *Ty-2* introgression line. The full-length coding sequence of the *Ty-2* gene was amplified with primers Ty-2-pDONR221-attB1 and Ty-2-pDONR221-attB2 (**Supplementary Table S2**) and cloned using the Gateway^®^ system into pDONR™221 vector (Invitrogen), and subsequently recombined to the binary vector pK7WG2 (Invitrogen) containing the CaMV 35S promoter. The obtained overexpression construct was introduced into *A. tumefaciens* strain GV3101. The full-length cDNA of the *Ty-1* gene was amplified as described by Verlaan et al. (2013) and cloned into pK7WG2 vector. Afterwards, this vector was electroporated into *A. tumefaciens* strain AGL1 + VirG.

The TYLCV ORFs *Rep*/*C1*, *C4*, and *V1* were cloned from TYLCV-IL [Almeria] (GenBank accession AJ489258) into Gateway^®^ donor vector pDONR™207 (Invitrogen) then also transferred to destination vector pK7WG2. The TYLCSV *Rep*/*C1* gene was cloned from TYLCSV (GenBank accession NC_003828) into pDONR™221 vector. vectors were transferred to *A. tumefaciens* strain GV3101. All primer sequences used for cloning are shown in **Supplementary Table S2**.

Amino acid sequences from the *Rep*/*C1* genes of the TYLCV-IL_Alm (GenBank accession number NP_658995), TYLCV-[CN:SH2] (CAK54965), TYLCSV used in Wageningen (designated TYLCSV-Wag; NP_620741), TYLCSV strains from Spain described by Díaz-Pendón et al. (2019; AGT57796 and P38609) and TYLCV-Mld strains described by Ohnishi et al. (2016; AFQ60612) and Díaz-Pendón et al. (2019; NP_786880) were analyzed by multiple sequence alignment using the CLUSTALW algorithm of MegAlign (DNA Star).

### *Agrobacterium* Transient Transformation Assay

In order to perform (co)-infiltration experiments by *Agrobacterium* transient transformation assay (ATTA) two preliminary tests were done to set up the system. Afterwards, three repeated experiments were carried out to obtain and confirm the results. The ATTA experiments were performed on 3-weeks-old *N. benthamiana* Domin plants with nine replicates based on a protocol by Van der Hoorn et al. (2000). Briefly, cultures of *A. tumefaciens* GV3101 and AGL1+virG from glycerol stocks of the previously described constructs were grown overnight at 28°C in 3 ml Luria-Bertani (LB) medium with antibiotics (20 µg/ml spectinomycin and 250 µg/ml rifampicin). The next day the OD_600_ was measured, the bacterial concentration was calculated and diluted with 15 ml yeast extract beef (YEB) medium (for 1 L: 5 g beef extract, 5 g bacteriological peptone, 1 g yeast extract, 5 g sucrose, 2 ml of 1 M MgSO4) with 1.5 µl 200 mM acetosyringone, 150 µl 1 M 2-(N-morpholino) ethanesulfonic acid (MES) (pH 5.6), and antibiotics (20 µg/ml spectinomycin and 250 µg/ml rifampicin). After overnight incubation at 28°C, cells were pelleted by centrifugation at 4,000 rpm for 10 min. The pellets were dissolved in 10 ml freshly made infiltration medium [for 1 L: 20 g sucrose, 5 g base MS salts (Duchefa Biochemie), 1.95 g MES (pH 5.6); 1 ml 200 mM acetosyringone was added]. The OD_600_ of the dissolved pellets was measured and adjusted to 2.

Agroinfiltration was performed on young, fully expanded *N. benthamiana* leaves. All single infiltrations were performed at OD_600_ = 2, while co-infiltrations were performed using a 1:1 mixture with a final OD_600_ of 1 for each construct. As a positive control an *Agrobacterium* strain containing the INF1 was infiltrated. INF1 is an elicitin protein secreted by the late blight pathogen *Phytophthora infestans* (Mont.) de Bary which is recognized by a cell surface receptor-like protein (RLP) in *N. benthamiana*, triggering a cell death-related immune response (Kamoun et al., 1998).

For expression analysis of the overexpressed genes in *N. benthamiana*, all the overexpression constructs were infiltrated individually in the top three fully expanded leaves of five independent plants. The INF1 construct was infiltrated as a positive control, and a mock-infiltration was performed as a negative control. The leaf disks covering the infiltrated areas were collected at 3 days after infiltration. From each plant the leaf disks infiltrated with the same construct were mixed together as one biological sample. Thus, per gene five biological samples were obtained. Total RNA was isolated using RNeasy Mini Kit (Qiagen). cDNA was produced using the iScript™ cDNA synthesis kit (Bio-Rad). Quantitative reverse-transcriptase PCR (RT-qPCR) was performed using iQ™ SYBR^®^ Green Supermix (Bio-Rad). The primer sequences are shown in **Supplementary Table S2**. For the mock-treated samples non-detect Ct values were replaced with an average of the detected (high) Ct values or were converted to 40 when the majority were non-detects (McCall et al., 2014). The constitutively expressed gene *NbL23* from *N. benthamiana* encoding a 60S ribosomal protein was used as reference gene (Liu et al., 2012). Relative expression level was calculated using the 2^−ΔΔCT^ method (Livak and Schmittgen, 2001). The expression data were normalized to *NbL23* and relative to the mock-infiltration. In addition, the RT-qPCR products were examined *via* 1.5% agarose gel electrophoresis. The experiment and analysis were repeated one more time to confirm the results.

### Visual Scoring of Hypersensitive Response

Four days post infiltration the leaves were scored by eye for visible hypersensitive response (HR) based on cell death incidence for the infiltrated spots (Rietman, 2011). Scoring was done with the help of a scale ranging from 0 (no cell death), 25, 50, 75, to 100% necrosis of the infiltrated area.

### 3,3’-Diaminobenzidine Staining

To detect hydrogen peroxide (H_2_O_2_) accumulation, 3,3’-diaminobenzidine (DAB) staining was carried out using an adaptation of previous methods (Thordal-Christensen et al., 1997; Daudi et al., 2012). A 10 mM Na_2_HPO_4_ DAB staining solution was generated by adding 0.05% v/v Tween 20 and 200 mM Na_2_HPO_4_ to 1 mg/ml DAB solution. The agroinfiltrated leaves were immersed in the DAB staining solution and gentle vacuum infiltration was performed for 5 min, following by incubation at 80 rpm shaking speed for 4 hours. Afterwards, the DAB staining solution was removed and replaced with bleaching solution ethanol:acetic acid:glycerol in a 3:1:1 ratio. The samples were heated till 90–95°C for 15 min, after which fresh ethanol:acetic acid:glycerol solution was added, and the leaves were incubated for another 30 min. Photographs were made on a plain white background under uniform lighting. DAB staining intensity indicating H_2_O_2_ levels was quantified by ImageJ. An arbitrary scale was used to show the results. Relative intensities were calculated by subtracting background scores from the scores of infiltrated areas.

## Results

### Fine Mapping of the *Ty-2* Gene Using Crosses With Susceptible *Solanum habrochaites*

Previously, we showed that the *Ty-2* gene from *S. habrochaites* is located in a region with suppressed recombination caused by a 200-kb inversion when compared with cultivated tomato *S. lycopersicum* (Wolters et al., 2015). In our quest to clone the *Ty-2* gene, a fine-mapping strategy was deployed to allow normal recombination by using progeny from crosses between the *Ty-2* line and three different TYLCV-susceptible *S. habrochaites* accessions (G1.1560, G1.1257, and G1.1290) (Wolters et al., 2015). F_1_ plants from G1.1257 were inter-crossed to produce pseudo-F_2_ progeny. BC_1_ families were obtained by backcrossing F_1_ plants from all three susceptible *S. habrochaites* parents to the (homozygous) *Ty-2* line.

**Figure 1:**
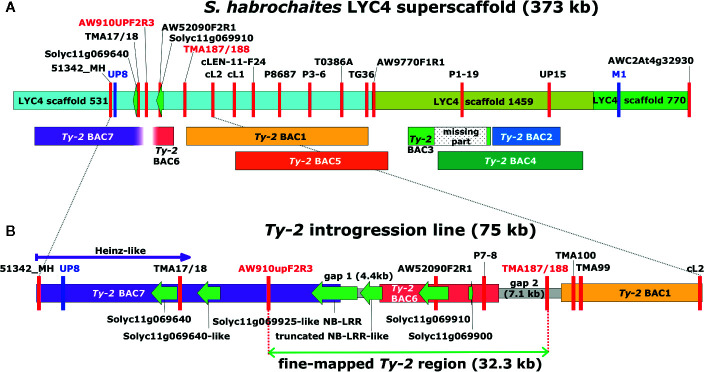
Graphical representation of the *Ty-2* genomic region in *Solanum habrochaites*. **(A)** The *S. habrochaites* LYC4 superscaffold was constructed from three scaffold sequences shown in different colors (Wolters et al., 2015). Markers are indicated with vertical red or blue lines. Markers UP8 and M1 in blue were previously reported to flank the 300-kb *Ty-2* region (Yang et al., 2014). Sequences homologous to genes Solyc11g069640 and Solyc11g069910 are indicated with green arrows. These flank the upper inversion breakpoint compared with the *Solanum lycopersicum* genome sequence. Bacterial artificial chromosome (BAC) clones containing homologous sequences from *Ty-2* introgression lines are shown below the LYC4 superscaffold. *Ty-2* BAC1-4 were obtained from the first BAC library and BAC5-7 from the second BAC library. The lower end of BAC7 and the upper end of BAC6 contained sequences without homology to the LYC4 superscaffold. **(B)** Representation of the full-length genomic sequence of the *Ty-2* line between markers 51342_MH and cL2. Sequences of gaps between BAC clones were obtained by long-range PCR and genome walking. Predicted genes showing homology to *S. lycopersicum* genes are indicated with green arrows. The 32.3-kb fine-mapped *Ty-2* region between markers AW910upF2R3 and TMA187/188 contains four predicted genes.

In the first round of recombinant screening, 88 pseudo-F_2_ plants and 415 BC_1_ plants were tested with markers 51342_MH and AWC2At4g32930 flanking the inverted region (**Figure 1**) to identify recombinants. From this screening, 10 pseudo-F_2_ and 22 BC_1_ recombinant plants were selected (**Supplementary Table S3**). Additional markers (TMA17/18, AW910upF2R3, AW52090F2R1, and TMA99) were developed showing polymorphisms between the *Ty-2* line and the *S. habrochaites* accessions in order to characterize the breakpoints of these recombinants (**Supplementary Table S4**). In addition, the F_2_ family was phenotyped for TYLCV resistance. As segregation of dominant resistance in the BC_1_ plants was not expected, no TYLCV disease assay was performed on these plants. From the recombinants, F_2_ recombinant A5 was susceptible to TYLCV, indicating that *Ty-2* is located below marker AW910upF2R3 (**Table 1**). All other recombinants were resistant. Recombination in plant D11 had occurred between markers P8687 and T0386A. Together, these results suggested that the *Ty-2* gene was present in the region between markers AW910upF2R3 and T0386A (**Table 1**). Based on the *S. habrochaites* accession LYC4 super scaffold assembly (The 100 Tomato Genome Sequencing Consortium, 2014; Wolters et al., 2015) this was a region of 108-kb.

**Table 1:** Recombinant screening and tomato yellow leaf curl virus (TYLCV) disease scores of pseudo-F_2_ progeny plants derived from a cross between *Ty-2* introgression line and *Solanum habrochaites* accession G1.1257.

F_2_ recombinant	Marker genotype/physical position (Mbp)^a^													DSI score 40 dpi (phenotype)^b^
	51342_MH	TMA17/18	*AW910 upF2R3*	AW52090F2R1	TMA99	cL2	cLEN-11-F24	P8687	*T0386A*	P1-19	UP15	M1	AWC2At4g32930	
	54.26	54.27	*	54.52	─	54.49	54.47	54.45	54.42	54.35	54.30	54.56	54.60	
D5	B	B	B	B	B	B	B	B	B	B	B	B	H	0 (R)
C11	B	B	B	B	B	B	B	B	B	H	H	H	H	0 (R)
H10	B	H	H	H	H	H	H	H	H	H	H	H	H	0 (R)
A5	H	H	H	A	A	A	A	A	A	A	A	A	A	2 (S)
D11	H	H	H	H	H	H	H	H	A	A	A	A	A	0 (R)
G4	H	H	H	H	H	H	H	H	H	H	A	A	A	0 (R)
F3	H	H	H	H	H	H	H	H	H	H	H	H	A	0 (R)
C10	H	H	H	H	H	H	H	H	H	H	H	H	H	0 (R)
H7	H	B	B	B	B	B	B	B	B	B	B	B	B	0 (R)
F10	H	H	H	H	H	H	H	H	H	H	H	H	B	0 (R)

^a^A, homozygous for susceptible S. habrochaites G1.1257 allele; B, homozygous for S. habrochaites Ty-2 allele; H, heterozygous; italicized markers are flanking markers of the Ty-2 region; physical positions of the markers are based on tomato Heinz genome version SL2.50 chromosome 11; ^*^marker on unmapped scaffold (chromosome 0); ─ S. habrochaites-specific marker; ^b^DSI, Disease Severity Index; dpi, days post inoculation; R, resistant; S, susceptible.

In the second stage of fine mapping, disease tests were performed using the BC_1_S_1_ progeny from BC_1_ recombinants. The BC_1_S_1_ family generated from BC_1_ recombinant 44–79 was particularly interesting since recombination in this plant had occurred between markers TMA99 and cL2 (**Supplementary Table S4**). All BC_1_S_1_ progeny plants from 44 to 79 were resistant against TYLCV (**Table 2**), indicating that *Ty-2* was located above marker cL2 in the region homozygous for the allele of the *Ty-2* introgression line (B). As a result, the location of *Ty-2* was narrowed down to a region between markers AW910upF2R3 and cL2, which was 38-kb in size according to the *S. habrochaites* LYC4 assembly.

**Table 2:** Genotypic and phenotypic analysis of BC_1_S_1_ population derived from BC_1_ recombinant PV155044-79.

BC_1_Recombinant	Marker genotype^a^							BC_1_S_1_ progeny^b^
	TMA17/18	AW52090F2R1	TMA99	*cL2*	cLEN-11-F24	P8687	T0386A	
PV155044-79	B	B	B	H	H	H	H	all TYLCV R (DSI 0)

^a^B, homozygous for S. habrochaites Ty-2 allele; H, heterozygous. Italicized marker is new flanking marker of the Ty-2 region.

^b^DSI, Disease Severity Index; R, resistant.

To trim the *Ty-2* region further in size, 537 plants from different F_2_ populations, derived from crosses with all three *S. habrochaites* accessions, were genotyped (**Supplementary Table S5**) with markers 51342_MH and AWC2At4g32930 flanking the *Ty-2* region. This resulted in the identification of 70 recombinants. Subsequently, these recombinants were genotyped throughout the whole region to identify the recombination sites. In addition, they were tested for TYLCV resistance. All recombinants showing marker scores B and H were resistant, as expected. The results for 30 recombinants showing markers scores A and H are shown in **Table 3**. Interestingly, the recombination breakpoint of plant 68–85 was between markers P7-8 and TMA187/188 and this plant was susceptible to TYLCV (**Table 3**). Therefore, the *Ty-2* gene was mapped above marker TMA187/188. When all results were taken together, the *Ty-2* region was fine-mapped to a region of approximately 21-kb (based on the *S. habrochaites* LYC4 assembly) flanked by markers AW910upF2R3 and TMA187/188 (**Figure 1**). Within the newly defined *Ty-2* region, only two putative genes homologous to Heinz Solyc11g069900 (alpha/beta-Hydrolases superfamily protein) and Solyc11g069910 (encoding a DNA-directed RNA polymerase II subunit RPB11-like protein) genes were present in the *S. habrochaites* LYC4 superscaffold assembly (**Figure 1**).

**Table 3:** Analysis of recombination breakpoints and tomato yellow leaf curl virus (TYLCV) disease scores of F_2_ progeny plants representing different recombination events.

Susceptible *S. habro-chaites* parent	F_2_ recombinant (s)	No. of recom-binants	Marker genotype/physical position (Mbp)^a^																
			51342_MH	TMA17/18	*AW910upF2R3*	AW 52090F2R1	P7-8	*TMA 187/188*	TMA100	TMA99	cL2	cL1	cLEN-11-F24	P8687	P3-6	T0386A	P1-19	AWC2At4g 32930	DSI score 42 dpi (phenotype)^b^
			54.26	54.27	*	54.52	54.52	54.51	54.51	─	54.49	54.48	54.47	54.45	54.44	54.42	54.35	54.60	
G1.1560	68-F12	1	A	nd	H	H	H	H	H	H	H	H	H	H	H	nd	nd	nd	0 (R)
G1.1560	68-85	1	A	A	A	A	A	H	H	H	H	H	H	H	H	H	H	H	4 (S)
G1.1560	69-101	1	A	A	A	A	A	A	H	H	H	H	H	H	H	nd	H	H	4 (S)
G1.1560	69-82	1	A	A	A	A	A	nd	A	A	H	H	H	H	H	H	H	H	3 (S)
G1.1560	69-41	1	A	A	A	A	nd	nd	A	A	A	A	nd	H	H	H	H	H	1.5 (S)
G1.1560	69-103/68-87	2	A	A	A	A	nd	nd	A	A	A	nd	A	A	A	A	H	H	4 (S)
G1.1290	74-35	1	A	A	A	A	nd	nd	nd	A	A	nd	A	A	A	A	H	H	2 (S)
G1.1560	70-41	1	A	A	A	A	nd	nd	nd	nd	nd	nd	A	A	A	A	H	H	3 (S)
G1.1560	68-83	1	A	A	A	A	nd	nd	nd	nd	A	nd	A	A	A	A	A	H	2 (S)
G1.1560	69-110/69-88	2	A	A	A	A	nd	nd	nd	nd	A	nd	nd	A	A	A	A	H	4 (S)
G1.1257	80-3	1	A	A	A	A	nd	nd	nd	nd	A	nd	A	A	A	A	A	H	3 (S)
G1.1257	80-4	1	A	A	A	A	nd	nd	nd	nd	A	nd	A	A	A	A	A	H	4 (S)
G1.1560	69-78	1	nd	A	A	A	nd	nd	nd	nd	nd	nd	nd	nd	A	nd	A	H	3 (S)
G1.1560	69-49	1	H	A	A	A	nd	nd	nd	nd	nd	nd	A	A	A	A	A	A	4 (S)
G1.1290	72-9	1	H	H	H	H	nd	nd	nd	H	H	nd	nd	nd	nd	A	nd	A	0 (R)
G1.1560	68-161/69-64	2	H	H	H	H	H	H	nd	nd	nd	nd	nd	nd	H	A	A	A	0 (R)
G1.1560	70-3/81-11	2	H	H	H	H	nd	nd	nd	nd	H	nd	nd	H	nd	H	nd	H	H	A	A	0 (R)
G1.1560	68-92/68-143/69-138/69-94	4	H	H	H	H	nd	nd	nd	H	nd	H	H	H	H	H	A	0 (R)
G1.1290	74-2/74-3/74-13	3	H	H	H	H	nd	nd	nd	H	H	nd	H	H	H	H	H	A	0 (R)
G1.1257	80-2	1	H	H	H	H	nd	nd	nd	H	H	nd	H	H	H	H	H	A	0 (R)

^a^A, homozygous for susceptible S. habrochaites allele; B, homozygous for S. habrochaites Ty-2 allele; H, heterozygous; nd, not determined italicized markers are flanking markers of the Ty-2 region; physical positions of the markers are based on the tomato Heinz genome version SL2.50 chromosome 11.

^*^marker on unmapped scaffold (chromosome 0); ─ S. habrochaites-specific marker.

^b^DSI score, Disease Severity Index; dpi, days post inoculation; R, resistant; S, susceptible.

### Cloning and Sequencing of the *Ty-2* Region

As *S. habrochaites* accession LYC4 is different from the source of the *Ty-2* gene *S. habrochaites* B6013, and furthermore susceptible to TYLCV, the full genomic sequence of the fine-mapped *Ty-2* region from the *Ty-2* introgression line had to be elucidated. To this end, a *Hin*dIII BAC library with 10x genome coverage was screened (Wolters et al., 2015) and yielded four BAC clones (partially) covering the *S. habrochaites* LYC4 super scaffold (*Ty-2* BAC 1-4, **Figure 1A**). From those, *Ty-2* BAC4 contained the lower inversion breakpoint, when compared to *S. lycopersicum* (Wolters et al., 2015). To close the gaps between the BAC clones, and to obtain BAC clones spanning the entire region between marker 53142_MH and the end of *Ty-2* BAC1, a new high coverage (35x) BAC library was generated. It proved difficult to obtain a stable BAC clone containing a large insert with marker AW52090F2R1 (**Figure 1**), but in the end one BAC clone with a small insert (BAC6) was obtained. This BAC clone was sequenced and shown to contain an insert of 13.4-kb. The clone contained marker P7-8, besides AW52090F2R1, but was negative for marker AW910upF2R3. The insert contained sequences homologous to Solyc11g069900 and Solyc11g069910, and a partial NLR type gene with homology to Solyc11g069660 which was absent from the LYC4 assembly (**Figure 1**). To obtain sequences from the *Ty-2* introgression line to the left of BAC6 the 35x BAC library was screened with marker AW910upF2R3, which resulted in the isolation of BAC7. Upon testing with marker AW52090F2R1, this clone proved to be negative. The nucleotide sequence of clone BAC7 was determined and revealed a part of an NLR type gene homologous to Solyc11g069925 (**Figure 1B**).

The gaps between BACs were closed by long-range PCR and genome walking. Primers were designed based on previously obtained *Ty-2* sequences and on conserved sequences in this region between *S. lycopersicum* Heinz, *S. habrochaites* LYC4, and *S. pennellii* LA0716 (**Supplementary Table S6**). The gap between BAC6 and BAC1 proved to be 7.1-kb in the *Ty-2* line, while the gap between BAC7 and BAC6 was 4.4-kb (**Figure 1B**). After assembly of the full-length sequence from BAC7 to BAC1, the *Ty-2* region from the *Ty-2* introgression line flanked by markers AW910upF2R3 and TMA187/188, was 32.3-kb in size (**Figure 1B**).

### Identification of Candidate Genes

The 32.3-kb sequence was analyzed for the presence of candidate genes by comparison with Heinz genome assembly SL2.50 (ITAG2.4 gene annotation), SL3.0 (ITAG3.2 gene annotation), and by using the FGENESH and Augustus *de novo* gene prediction programs. This resulted in four candidate genes showing homology to Heinz genes Solyc11g069900 (alpha/beta-Hydrolases superfamily protein), Solyc11g069910 (DNA-directed RNA polymerase II subunit RPB11-like protein), Solyc11g069660 (partial NLR type gene), and Solyc11g069925 (full length NLR type gene). The latter was obtained from the ITAG3.2 annotation of genome Heinz version SL3.0. In Heinz version SL2.50 with ITAG2.5 annotation the predicted full-length gene was Solyc11g069930.

Sequences of the predicted genes and proteins were compared between the *Ty-2* line, susceptible *S. habrochaites* LYC4, and *S. lycopersicum* Heinz. Both coding sequence and protein sequence of the Solyc11g069900-like gene from the *Ty-2* line were identical to those from LYC4. The coding sequence of the Solyc11g069910-like gene from the *Ty-2* line contained 1 SNP compared with LYC4, but this was a synonymous SNP. However, the NLR-type genes were absent from the *S. habrochaites* LYC4 super scaffold. The partial gene from the *Ty-2* line is indicated as Solyc11g069660-like. The coding sequence of Solyc11g069660 is 2628-bp in Heinz (ITAG2.4) and only 756-bp in the *Ty-2* line. On the other hand, the full-length NLR gene is indicated as Solyc11g069925-like. Both mRNA and protein sequences showed many differences compared with the Heinz sequences. From all four genes the Solyc11g069925-like gene therefore seemed the most likely candidate for the *Ty-2* resistance gene. Comparison of genomic DNA sequences of the Solyc11g069925-like gene with the published sequence of *TYNBS1* (GenBank accession LC126696) from Yamaguchi et al. (2018) showed that they were identical.

### Development of a *Ty-2* Specific Marker

For breeding purposes, a marker closely linked to and specific for the *Ty-2* gene is highly desirable. Considering that the *Ty-2* gene sequence shows a high level of homology to other NLR genes, both within the species and compared with other *Solanum* species, a marker based on the NLR sequence would likely not be specific enough for *Ty-2*. Therefore, we set out to develop flanking markers based on nearby unique sequences. From the three susceptible *S. habrochaites* accessions used for fine-mapping of *Ty-2*, two (G1.1257 and G1.1290) showed higher similarity to the *Ty-2* region introgressed from *S. habrochaites* B6013 than accessions G1.1560 and LYC4, as indicated by the presence of polymorphisms for the markers in **Supplementary Table S1**. Markers AW910upF2R3 and AW52090F2R1, flanking the *Ty-2* NLR gene on opposite sides, showed polymorphisms not only between the *Ty-2* line and *S. lycopersicum* Heinz (and MM), but also between the *Ty-2* line and *S. habrochaites* accessions G1.1257, G1.1290, and G1.1560. In addition, homologous sequences to these markers were identified from *S. habrochaites* LYC4, and other *Solanum* species like *Solanum pimpinellifolium* L., *Solanum arcanum* Peralta, and *S. pennellii* from the SGN database. Marker AW910upF2R3 is located at a distance of 4 kb proximal to the *Ty-2* NB-LRR gene. Primers of this marker amplified a 523-bp PCR product in the *Ty-2* line in contrast to a large PCR product of 821–839 bp in *S. lycopersicum* and the tested wild tomato accessions (**Supplementary Figure S1**). For *S. pennellii* LA0267 a 1,238-bp product is predicted (**Supplementary Table S7**). Presence of the 523-bp fragment was highly correlated with TYLCV resistance in the F2 and F3 progeny of crosses between the *Ty-2* line and susceptible *S. habrochaites* accessions in this study (**Supplementary Table S8**). Thus, marker AW910upF2R3 was shown to be a very useful SCAR marker to distinguish the (functional) *Ty-2* allele from those of all other mentioned accessions.

### Restricted Resistance Spectrum of *Ty-2*

**Figure 2:**
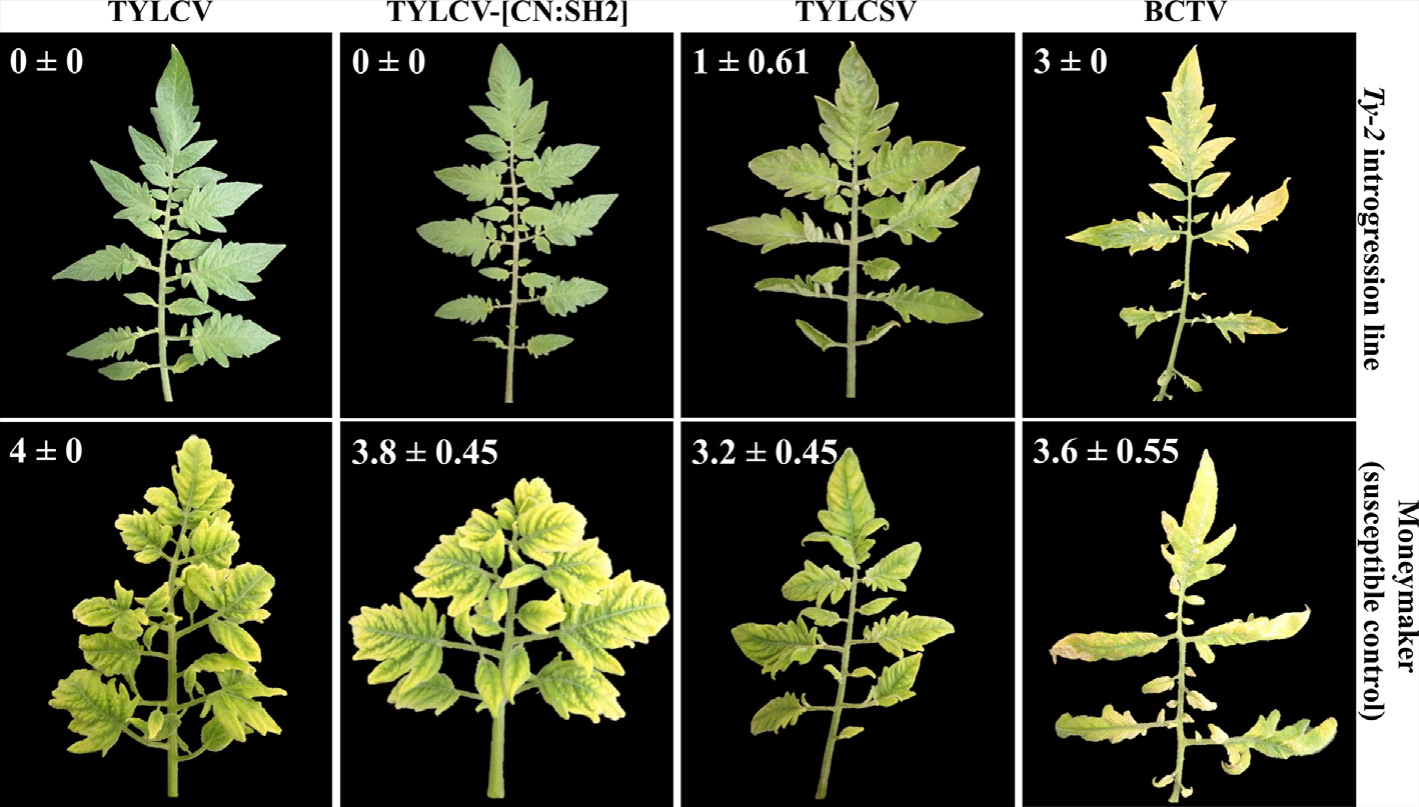
Disease reaction of the *Ty-2* introgression line and the susceptible control ‘Moneymaker’ to several tomato yellow leaf curl virus (TYLCV) strains and species: the Israel strain of TYLCV (TYLCV-IL), tomato yellow leaf curl Sardinia virus (TYLCSV), and tomato yellow leaf curl virus -[China: Shangai2] (TYLCV-[CN : SH2]) as well as beet curly top virus (BCTV) at 55 days post inoculation. Disease severity index (DSI) is described in *Materials and Methods*. Results are presented as mean ± standard deviation.

**Figure 3:**
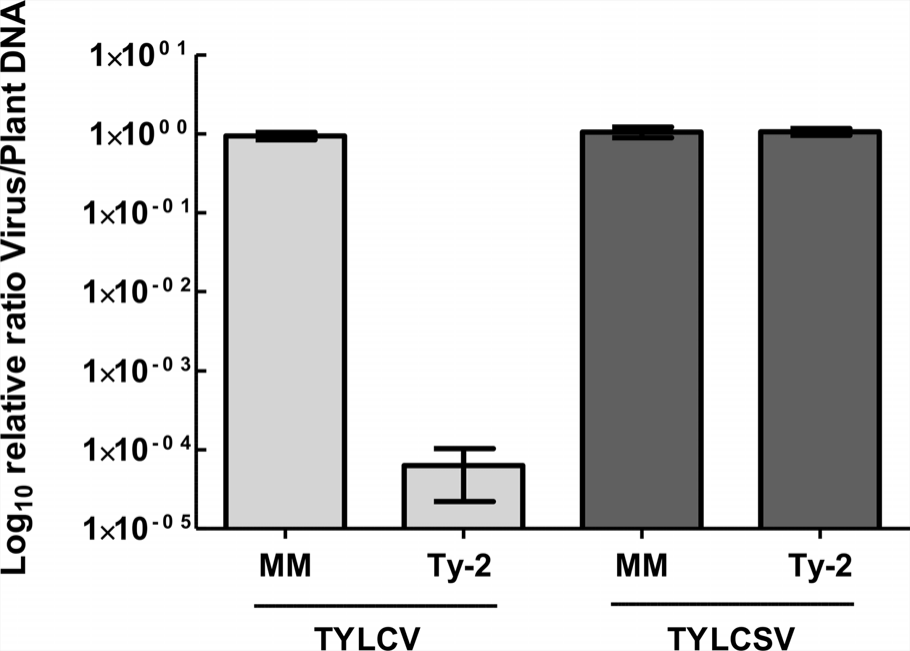
Relative tomato yellow leaf curl virus (TYLCV) and tomato yellow leaf curl Sardinia virus (TYLCSV) accumulation of *Ty-2* introgression line compared with susceptible Moneymaker (MM) as determined by qPCR. Values were normalized relative to *elongation factor 1α* (*EF1α*), and referenced to the levels in MM plants (set at 1). Viral titer values are displayed on a logarithmic scale (log10). Error bars represent the standard deviation.

As *Ty-2* was shown to be an NLR-type resistance gene, the spectrum of resistance of this gene is likely narrow. To test whether the resistance conferred by *Ty-2* is restricted to particular TYLCD-causing viruses, the *Ty-2* introgression line was agroinoculated with infectious clones of various viral species, i.e., TYLCV-IL, TYLCSV, and tomato yellow leaf curl virus-[China : Shanghai2] TYLCV-[CN:SH2]). In addition, the *Ty-2* line was challenged with a completely distinct leafhopper-transmitted *Curtovirus*, *in casu* beet curly top virus (BCTV). BCTV is a geminivirus that has recently become a serious threat for tomato production in the Western USA. It has also been reported in Mexico and South America, the Middle East, and Mediterranean basin (Chen et al., 2010). While, as expected, the susceptible control *S. lycopersicum* cv. Moneymaker (MM) showed severe disease symptoms of all tested viral strains and species (**Figure 2**), the *Ty-2* introgression line exhibited a high level of resistance against TYLCV-IL (DSI = 0) and TYLCV-[CN:SH2] (DSI = 0). The latter virus strain shows more than 97% identity in nucleotide sequence compared with TYLCV-IL and is therefore considered as a variant of TYLCV-IL (Zhang et al., 2009). Subsequently, TYLCV-IL virus accumulation was determined in systemic leaves *via* quantitative PCR (qPCR). In susceptible MM plants TYLCV-IL titer was high (Ct values 12), while in the *Ty-2* introgression line viral accumulation was much lower (Ct values 30). The fold difference in titer between the susceptible MM plants and the *Ty-2* introgression line was calculated using the ΔΔCt method (Livak and Schmittgen, 2001), with the level of viral accumulation in MM set at 1 (**Figure 3**). Viral accumulation was more than 10,000 times lower in the *Ty-2* line compared to MM.

In contrast to TYLCV-IL and TYLCV-[CN:SH2], *Ty-2* was ineffective against TYLCSV, a distinct viral species of TYLCV (Abhary et al., 2007), as the *Ty-2* line showed moderate yellowing and curling symptoms (DSI ≥ 1) (**Figure 2**). To support this finding, relative viral titer of TYLCSV was determined by qPCR in both MM and the *Ty-2* introgression line. TYLCSV accumulation was clearly detected in *Ty-2* plants (Ct values 11) and the level was comparable to that of the susceptible MM plants (**Figure 3**). Furthermore, BCTV was also able to successfully establish a systemic infection in *Ty-2* carrying plants, as observed by the presence of severe disease symptoms (DSI ≥ 3) (**Figure 2**). Altogether, the results indicate that *Ty-2* confers resistance to a limited number of *Begomoviruses* involved in TYLCD.

### The Rep/C1 Protein Is a Likely Candidate for the Avr Determinant of *Ty-2*

Earlier studies by Ohnishi et al. (2016) pointed toward the genomic region with overlapping *Rep*/*C1* and *C4* genes, to encode the putative effector of *Ty-2* resistance. To test which of the two genes triggered *Ty-2* mediated resistance, the genes encoding Rep/C1, C4, and CP/V1 proteins of the resistance-inducing TYLCV-IL strain were cloned and transiently co-expressed with *Ty-2* in *N. benthamiana*. We chose not to perform agroinfiltration of viral genes in the *Ty-2* tomato line, because most *Agrobacterium* strains induce background necrosis in tomato (Wroblewski et al., 2005), which may interfere with the hypersensitive response (HR) triggered by interaction between Ty-2 and the viral effector protein. As a negative control for HR-induction, the *Ty-1* gene was included, which confers resistance based on a different mechanism than NLR-type genes (Verlaan et al., 2013; Butterbach et al., 2014). As a positive control, elicitin protein INF1 was included, whose recognition by a cell surface receptor-like protein in *N. benthamiana* triggers a programmed cell death response (Kamoun et al., 1998).

To investigate whether the cloned genes were expressed after infiltration in *N. benthamiana* leaves a RT-qPCR was performed, including mock-treated leaves as control, and using the *N. benthamiana* gene *NbL23* as reference gene. The results shown in **Supplementary Figure S2** indicate that viral genes *V1*, *Rep/C1* and *C4* of TYLCV-IL, *Rep/C1* of TYLCSV, and the tomato *Ty-2* gene were all expressed at high levels in infiltrated leaves. These results were confirmed in a second experiment.

**Figure 4:**
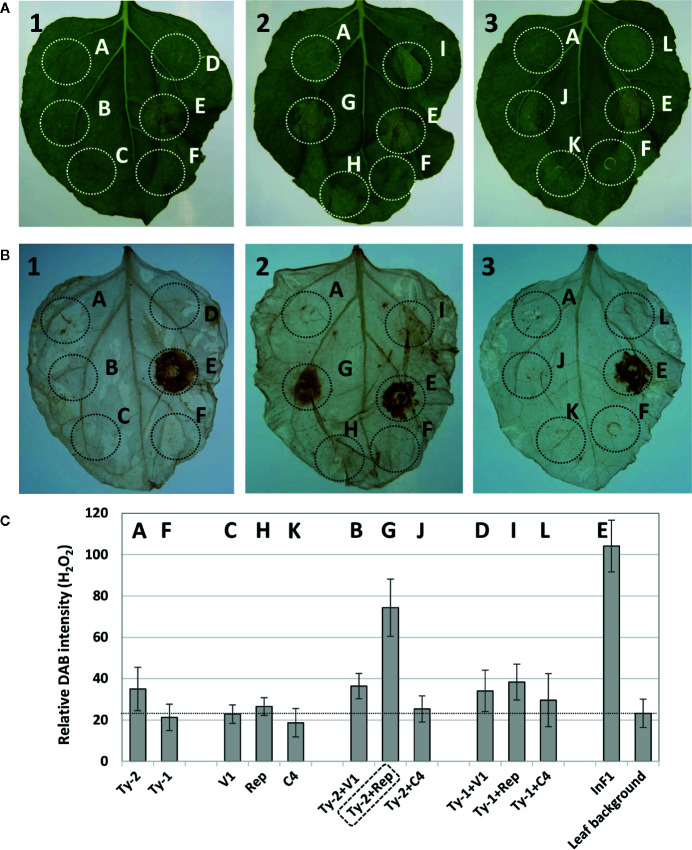
Hypersensitive response after co-infiltration of *Ty-2* gene with tomato yellow leaf curl virus (TYLCV) genes. **(A)** Pictures of *Nicotiana benthamiana* leaves co-infiltrated with constructs expressing the *Ty-2* or *Ty-1* gene of tomato with *CP*/*V1*, *Rep*/*C1*, or *C4* of TYLCV-IL, compared with infiltration of positive control INF1. **(B)** DAB staining of the same leaves as shown in **(A)**. Leaf 1 shows the co-infiltration with the viral gene *CP*/*V1*, leaf 2 with *Rep*/*C1* and leaf 3 with *C4*. The letters correspond to the following samples: spot A=*Ty-2*, B= *Ty-2*+ *CP*/*V1*, C= *CP*/*V1*, D=*Ty-1*+ *CP*/*V1*, E=INF1, F=*Ty-1*, G=*Ty-2*+ *Rep*/*C1*, H= *Rep*/*C1*, I=*Ty-1*+ *Rep*/*C1*, J=*Ty-2*+*C4*, K=*C4*, L=*Ty-1*+*C4*. Note the hypersensitive response (HR) response for spot G=*Ty-2*+ *Rep*/*C1*. **(C)** Quantification of 3,3’-diaminobenzidine (DAB) staining. Values are means ± SD from three independent experiments.

In total, three co-expression experiments were performed to obtain and confirm the results. A representative picture of the obtained results is shown in **Figure 4A**. The leaves showed a clear HR necrotic area with the positive control INF1 (spot E), whereas none of the individual gene constructs (viral genes or *Ty-2*) triggered visible HR response. When viral genes were co-expressed with *Ty-2*, only the co-expression of *Ty-2* with the *Rep*/*C1* gene of TYLCV-IL consistently resulted in significant HR. The results of necrosis scoring are shown in **Supplementary Table S9**. While INF1 yielded a score of 50% necrosis (as compared with a very strong HR response upon co-infiltration of *Avr2* from *P. infestans* with the potato *R2* resistance gene (Rietman, 2011), *Ty-2* with *Rep*/*C1* resulted in a visual score of 14.5% necrosis.

DAB staining of the leaves was performed to detect H_2_O_2_ accumulation, another indication of HR. H_2_O_2_ accumulation was detected at spots where *Ty-2* was co-expressed with the *Rep*/*C1* gene of TYLCV-IL (**Figure 4B**). The intensity was comparable with the positive control (**Figure 4C**) and indicated that co-expression of *Ty-2* and *Rep*/*C1* of TYLCV-IL, but not *C4*, resulted in reactive oxygen species accumulation. To further substantiate these findings, the experiment was repeated but now also using *Rep*/*C1* constructs from TYLCSV (**Supplementary Figure S3A**), to which *Ty-2* does not confer resistance. The results showed that *Rep*/*C1* of TYLCV, but not of TYLCSV, led to H_2_O_2_ accumulation when co-expressed with *Ty-2* (**Supplementary Figures S3B, C**).

### Amino Acid Sequence Alignment of *Rep*/*C1* From Resistance-Inducing and -Breaking Strains

**Figure 5:**
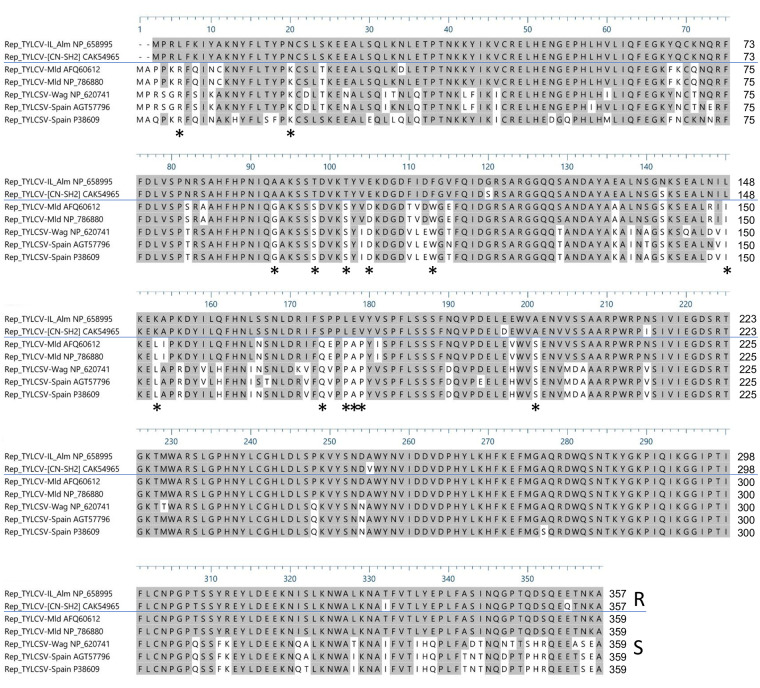
Alignment of Rep/C1 protein sequences of resistance-inducing [tomato yellow leaf curl virus (TYLCV)-IL-Alm and TYLCV-[CN:SH2]] and resistance-breaking [tomato yellow leaf curl Sardinia virus (TYLCSV) and TYLCV-Mld] strains. Rep/C1 protein sequences of TYLCV-IL-Alm and TYLCSV-Wag are represented by GenBank accessions NP_658995 and NP_620741, respectively. Rep/C1 protein sequences of TYLCV-[CN : SH2] (GenBank accession CAK54965), TYLCV-Mld [JR : Kis] (AFQ60612), TYLCV-Mld Es-72-97 (NP_786880), TYLCSV-Es-Mur-TY2-Tom-11 (AGT57796), and TYLCSV-Es-Mur1-92 (P38609) were obtained from the NCBI database. Differences between resistance-inducing strains (R) and resistance-breaking strains (S) are indicated with asterisks beneath the alignment.

To identify possibly important amino acid residues in Rep/C1 needed for *Ty-2*-mediated resistance triggering, amino acid sequences of Rep/C1 of resistance-inducing strains TYLCV-IL and TYLCV-[CN:SH2] and resistance-breaking strains of TYLCSV and TYLCV-Mld (Ohnishi et al., 2016; Díaz-Pendón et al., 2019) were aligned. Prior to this, the *Rep*/*C1* gene of TYLCV-IL and TYLCSV were cloned and their nucleotide sequences were obtained. Subsequently, the protein sequences were determined. These proved to be the same as GenBank accession numbers NP_658995 (TYLCV-Il-Alm) and NP_620741, respectively. Simultaneously, Rep/C1 protein sequences of TYLCV-[CN:SH2] (GenBank accession number CAK54965) and TYLCV-Mld [JR : Kis] (Ohnishi et al., 2016; GenBank accession number AFQ60612) were retrieved from the NCBI database. In addition, Rep/C1 protein sequences from other viral strains reported to infect *Ty-2*-containing lines (Díaz-Pendón et al., 2019) were retrieved from NCBI [TYLCV-Mld Es-72-97 (NP_786880), TYLCSV-Es-Mur-TY2-Tom-11 (AGT57796), and TYLCSV-Es-Mur1-92 (P38609)]. A multiple sequence alignment revealed 27 amino acid changes that were different between resistance-inducing and all resistance-breaking strains, 14 of which were conserved in the resistance-breaking strains (**Figure 5**, indicated by asterisks).

## Discussion

The *Ty-2* gene in tomato conferring resistance to TYLCV has been extensively exploited in tomato breeding worldwide, in combination with other *Ty* genes (mostly *Ty-1*/*Ty-3* or *ty-5*) (Yan et al., 2018). In this study, we have described our successful positional cloning approach to isolating the *Ty-2* gene. The gene presents an NLR-type of resistance gene. Using agroinfiltration to transiently co-express *Ty-2* with the *Rep*/*C1* and *C4* gene of resistance-inducing strain (TYLCV-IL) and resistance-breaking strain (TYLCSV) respectively in *N. benthamiana*, only the Rep/C1 protein of TYLCV consistently triggered a hypersensitive response when co-expressed with the *Ty-2* gene, indicating that this protein most likely presents the Avr determinant of *Ty-2*-based (strain-specific) resistance response.

### *Ty-2* Cloning Approaches

Successful cloning of the *Ty-2* gene has long been hampered by the occurrence of suppression of recombination in the *Ty-2* introgressed region from *S. habrochaites* into cultivated tomato (Yang et al., 2014), caused by a chromosomal inversion (Wolters et al., 2015). In this study we have shown that this problem can be circumvented by crossing the *Ty-2* introgression line with TYLCV-susceptible *S. habrochaites* accessions, and providing informative recombinants. During our long efforts to clone *Ty-2*, this approach finally allowed us to fine-map *Ty-2* to a small region containing a few candidate genes, among which one NB-LRR gene that turned out to be the TYLCV resistance gene. Meanwhile, and in parallel to our studies, Yamaguchi et al. (2018) sequenced the inversion breakpoints and identified two NB-LRR genes, one of which was subsequently shown to confer TYLCV resistance. While both approaches have been successful, the one as described in this paper is more commonly applied and unbiased, and especially useful when an unknown, non-NB-LRR gene turns out to present the resistance candidate gene.

### *Ty-2* Resistance Mechanism

The resistance provided by NLR genes results in absence of disease symptoms and in most cases, no pathogen can be detected beyond the primary infection site (Boualem et al., 2016). Several studies have reported that restriction of TYLCV infection by *Ty-2* leads to absence of detectable viral DNA in systemic tissues (Shahid et al., 2013; Bai et al., 2016; Yamaguchi et al., 2018). However, in our experimental conditions *Ty-2* does not completely restrict TYLCV replication because viral DNA can be detected beyond the site of inoculation. A low level of TYLCV DNA in challenged *Ty-2* plants has also been reported by Barbieri et al. (2010); Li et al. (2016) and Tabein et al. (2017). These findings are further supported by the detection of TYLCV-specific small interfering RNAs (siRNAs) in systemic tissues, which indicates that *Ty-2*-carrying lines do not entirely halt virus replication (Butterbach et al., 2014).

The most common disease defense response activated by NLR genes is the induction of localized programmed cell death (HR) (Boualem et al., 2016; Gouveia et al., 2017). However, NLR-mediated resistance toward viruses is not always associated with HR (de Ronde et al., 2014a; Boualem et al., 2016). An example is the extreme resistance to potato virus X (PVX) conferred by potato NLR gene *Rx1* (Bendahmane et al., 1999). The *Rx1* gene is triggered by the PVX capsid protein, but the underlying resistance mechanism is different from HR/cell death response. Similarly, in this study, resistance mediated by *Ty-2* did not reveal a (clear visual) HR in the tomato introgression line. This can be explained by the fact that *Begomoviruses* are restricted to the phloem, and in case an HR would occur in resistant plants it would not be visually detectable in the leaves. This hampered the identification of the Avr determinant of *Ty-2*. However, HR was observed when the *Ty-2* gene was transiently co-expressed with the *Rep*/*C1* viral gene in *N. benthamiana*. Further experiments are needed to verify whether HR occurs in the *Ty-2* tomato plants when challenged with an avirulent TYLCV strain.

*Ty-2* is a CC-NBS-LRR (CNL) type of gene belonging to the *I-2* like subclass of CNL genes. *I2*-like genes in tomato and potato show high divergence (Wei et al., 2014) and distinctly segregates in sequence and function from other CNLs (Bentham et al., 2018). The resistance mechanism of this subclass of CNLs is unknown (Kourelis and van der Hoorn, 2018).

In general, a direct or indirect perception of an effector by NLR genes triggers plant defense responses involving numerous downstream signaling pathways, such as mitogen-activated protein kinase (MAPK) signaling cascades, phytohormones involved in defense, and a set of defense-related genes (e.g. WRKY transcriptional factors) (Elmore et al., 2011; de Ronde et al., 2014a; Boualem et al., 2016). Previously, genome-wide detection of differentially expressed genes (DEGs) using a resistant *Ty-2* line and a susceptible control genotype has been reported (Chen et al., 2013). In this latter work, TYLCV-inoculated samples were harvested from 3, 5, and 7 days post inoculation (DPI) and were mixed for further analysis. A large number of preferentially expressed genes in the *Ty-2-*carrying line was then identified including WRKY transcriptional factors, protein kinases and receptor like kinases (Chen et al., 2013). However, their involvement in *Ty-2* resistance still remains elusive.

### The Rep/C1 Protein Is a Determinant of Systemic Infection of *Ty-2* Plants

The TYLCV genome has a large potential to change due to high recombination frequency which greatly enhances the genetic diversification of TYLCV populations (Seal et al., 2006; Abhary et al., 2007; Moriones and Navas-Castillo, 2008; Hosseinzadeh et al., 2014). To date, many natural *Begomoviruses* recombinants have been reported (Moriones et al., 2007). The ongoing evolution of new TYLCV variants by recombination poses a constant threat to the resistance conferred by an NLR gene. Some natural recombinant strains able to break *Ty-2* resistance have already been reported in tomato (Barbieri et al., 2010; Ohnishi et al., 2016). Our study indicated that the genetic determinant involved in overcoming *Ty-2-*mediated resistance is probably associated with the Rep/C1 protein. In contrast, Tomás et al. (2011) reported that the C4 protein is the viral factor associated with the ability of the TYLCV-Mld strain to systemically infect a *S. habrochaites* accession that is resistant to the TYLCV-IL strain. However, they did not indicate whether the resistance to TYLCV-IL in the *S. habrochaites* accession was conferred by a *Ty-2* allele or by novel gene(s) (Tomás et al., 2011).

In many parts of the world recent outbreaks and/or epidemics of viral diseases have been proven to be associated with the emergence of recombinant strains (Abhary et al., 2007; Lefeuvre et al., 2007; Belabess et al., 2015). These strains contain different recombined genomes. The recombination sites are not distributed randomly along the genome. The highest frequency of recombination has been observed in the regions of *Rep*/*C1* and *C4* genes, and are referred to as recombination hot spots (Fauquet et al., 2005; Abhary et al., 2007; Lefeuvre et al., 2007; Prasanna and Rai, 2007). Recombinant viruses like tomato yellow leaf curl Málaga virus (TYLCMalV) and tomato yellow leaf curl Axarquía virus (TYLCAxV) have a broader host range than either of the parental strains and are responsible for the TYLCD epidemics in Spain (Navas-Castillo et al., 2011). In the case of *Ty-2*, diversifying selection of the effector protein Rep/C1 could have led to the emergence of resistance-breaking strains (Barbieri et al., 2010; Ohnishi et al., 2016).

Functional analyses of *Begomoviruses*-encoded proteins have suggested that Rep/C1 and C4 are essential for virus replication and systemic spreading *via* vascular tissue, respectively (Gutierrez, 2000; Rojas et al., 2001). They are multifunctional proteins and also play an important role in RNA silencing suppression (Castillo et al., 2003; Rodriguez-Negrete et al., 2013). Besides an important role in gene regulation and chromosome dynamics, RNA silencing also acts as an antiviral defense mechanism in plants that is suppressed by viral RNA silencing suppressor proteins, also regarded as pathogenicity factors (Pumplin and Voinnet, 2013). Rep/C1 and C4 proteins determine pathogenicity by counteracting host RNA silencing machinery. They can impair DNA methylation *via* downregulation of plant DNA methyltransferases Methyltransferase 1 (MET1) and chromomethylase 3 (CMT3) (Rodriguez-Negrete et al., 2013). Plant NLR gene-mediated resistance, triggered by effectors, is considered to be a second layer of defense. The co-evolution of viruses and plants with a key role for viral RNA silencing suppressor proteins as effectors suggests a link between RNA silencing and NLR-gene-mediated immunity. Several plant virus-encoded RNA silencing suppressors have previously been reported as Avr determinants. For example, the turnip crinkle virus (TCV) coat protein is the Avr determinant for NLR-type resistance protein HRT from *Arabidopsis* and acts as a RNA silencing suppressor (Moffett, 2009). The tomato spotted wilt virus (TSWV) NSs protein functions simultaneously as RNA silencing suppressor and Avr determinant of *Tsw* gene-mediated resistance (de Ronde et al., 2013). However, more recent studies on alanine substitution mutants of TSWV NSs have shown that its ability to suppress antiviral RNA interference (RNAi) can be uncoupled from its function as effector of the *Tsw* resistance gene (de Ronde et al., 2014b). In light of this, and the multifunctionality of viral proteins, it is not unlikely that for TYLCV Rep/C1 its ability to suppress antiviral RNAi might also be uncoupled from its function as effector.

We have shown that an HR response is triggered when *Ty-2* is co-expressed with the Rep/C1 protein of TYLCV. Therefore, this viral protein likely presents the Avr determinant of *Ty-2*. Multiple different amino acids are present in the Rep/C1 protein sequences of resistance-breaking TYLC(S)V strains compared to resistance-inducing TYLCV strains. The central part of Rep/C1, which approximately consists of amino acids 120 to 180, is known as a multitasking interactor domain (Fondong, 2013; Lucioli et al., 2014). Expression of the first 210 amino acids of TYLCSV Rep/C1, but not of only the first 130 amino acids, in tomato cultivar Moneymaker caused upregulation of genes related to the plant immune system and genes involved in the negative regulation of programmed cell death (Lucioli et al., 2016). It will be interesting to investigate whether this central part is also crucial for triggering of the *Ty-2* resistance response. For this, a set of Rep/C1 mutants can be generated by amino acid reversions in Rep/C1 from resistance-breaking strains in a resistance-inducing background and amino acid substitutions in Rep/C1 from resistance-inducing strains in a resistance-breaking background. Analyzing these mutants for their ability to trigger/overcome *Ty-2* resistance could point toward the causal amino acid residues.

## Conclusion

Fine-mapping and cloning of the *Ty-2* gene was successfully achieved using the strategy of crossing the *Ty-2* introgression line with susceptible *S. habrochaites* accessions. The Rep/C1 protein of TYLCV has been identified to trigger *Ty-2*. Simultaneously, a very useful marker that can be used in breeding programs aimed at introgression of the *Ty-2* gene in different genetic backgrounds has been developed.

## Data Availability Statement

The datasets presented in this study can be found in online repositories. The names of the repository/repositories and accession number(s) can be found in the article/supplementary material.

## Author Contributions

XS, ZY, YB, RK, and A-MW conceived and designed the research. XS, ZY, XW, YW, and MA performed the experiments and analyzed the data. XS, ZY, YB, and A-MW wrote the manuscript with important contributions from RK, RV, and YD. All authors contributed to the article and approved the submitted version.

## Funding

This project was funded by the Netherlands Top Consortium for Knowledge and Innovation (TKI project TU-370), China Scholarship Council-fellowship for XS, the National technology system of commodity vegetable industry (CARS-23-A10) and the National Natural Science Foundation of China (31372070) for XW and YD, the National Natural Science Foundation of China (31972424) for YW.

## Conflict of Interest

The authors declare that the research was conducted in the absence of any commercial or financial relationships that could be construed as a potential conflict of interest.

## Supplementary Material

Supplementary Figure S1 Gel pictures showing PCR products of SCAR marker AW910upF2R3 in the *Ty-2* line, susceptible *S. habrochaites* accessions used in this study, segregating F2 progeny and other tomato lines and wild tomato accessions.

Supplementary Figure S2 Expression of viral genes and *Ty-2* gene in mock-treated and infiltrated leaves of *Nicotiana benthamiana*. **(A)** RT-qPCR results showing relative expression of target genes using the *N. benthamiana* gene *NbL23* as reference. Values were normalized against mock control and displayed on a logarithmic scale (Log10). The bars show the average value from five biological samples. Error bars indicate the standard deviation. In addition, individual data points are shown. **(B)** Gel pictures of RT-qPCR products.

Supplementary Figure S3 Hypersensitive Response after co-infiltration of *Ty-2* gene with TYLCV or TYLCSV *Rep*/*C1* gene. **(A)** Set-up of the co-expression experiment in *N. benthamiana*. The experiment was repeated three times. In the first experiment ten plants were used, and three leaves per plant were agroinoculated. In the second and third experiment six plants were used. **(B)** Representative pictures of *N. benthamiana* leaves three days after infiltration. **(C)** Results of DAB staining of the same leaves as shown in **(B)**.

Supplementary Table S1 Molecular markers used for fine-mapping of *Ty-2* on tomato chromosome 11.

Supplementary Table S2 Primes used for co-expression experiments.

Supplementary Table S3 Recombinant screening of crosses between *Ty-2* introgression line and susceptible *S. habrochaites* accessions using flanking markers 51342_MH and AWC2At4g32930.

Supplementary Table S4 Marker analysis of BC_1_ recombinants derived from crosses between *Ty-2* introgression line and three susceptible *S. habrochaites* accessions.

Supplementary Table S5 Recombination events identified in F_2_ families derived from crosses between the *Ty-2* introgression line and three susceptible *S. habrochaites* accessions.

Supplementary Table S6 Primers used for genome walking or long-range PCR to close the gaps between *Ty-2* BAC7 and BAC6, and between BAC6 and BAC1.

Supplementary Table S7 SCAR marker AW910upF2R3. Size or expected size of the PCR product with primers AW910upF2 and AW910upR3 (**Supplementary Table S1**) for genotypes/accessions of several *Solanum* species.

Supplementary Table S8 Correlation of presence of 532-bp fragment of SCAR marker AW910upF2R3 with TYLCV resistance conferred by *Ty-2* gene.

Supplementary Table S9 Visual scoring of the HR response in co-infiltrated leaves. Scoring was performed at four days after infiltration. The letters correspond to the spots in **Figure 4**. Scoring was performed using a scale of 0-100%, where 0 indicates no HR and 100% indicates maximum HR (100% of infiltration area showing necrosis).
