# Supplementary figures and images for: The NLR Protein Encoded by the Resistance Gene Ty-2 Is Triggered by the Replication-Associated Protein Rep/C1 of Tomato Yellow Leaf Curl Virus
Source: Front Plant Sci. 2020 Sep 10;11:545306. doi: 10.3389/fpls.2020.545306 (PMC7511541; doi:10.3389/fpls.2020.545306)

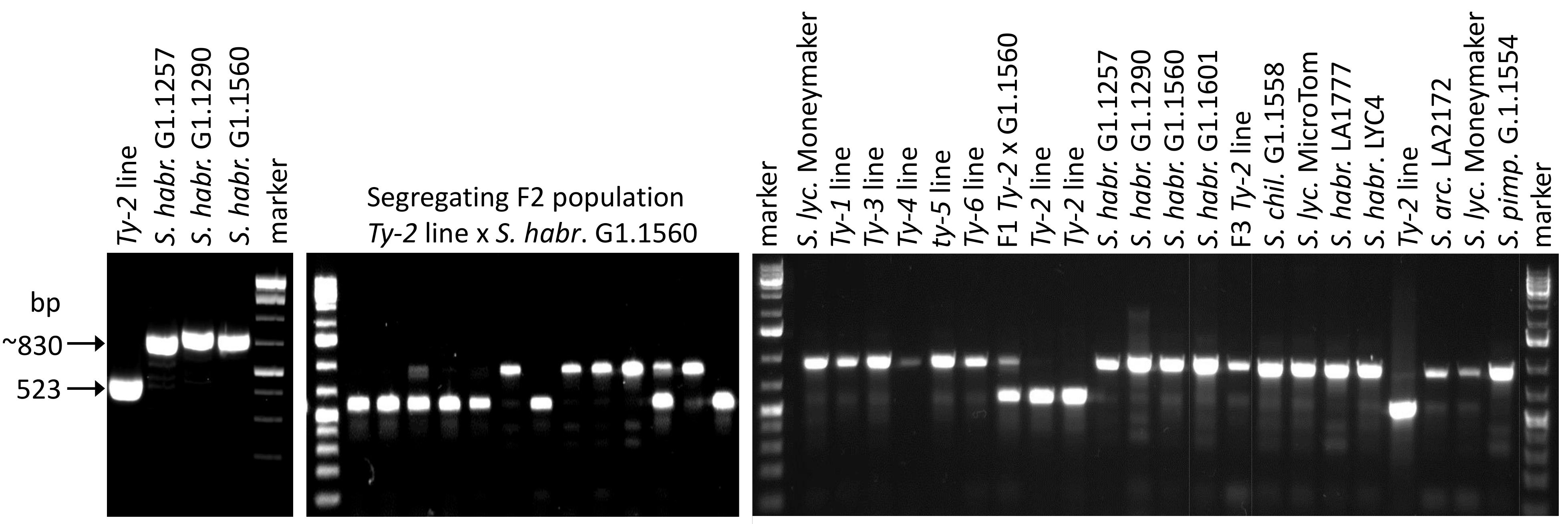

Supplement: Supplementary Figure S1 — Gel pictures showing PCR products of SCAR marker AW910upF2R3 in the Ty-2 line, susceptible S. habrochaites accessions used in this study, segregating F2 progeny and other tomato lines and wild tomato accessions. [file Image_1.tif]

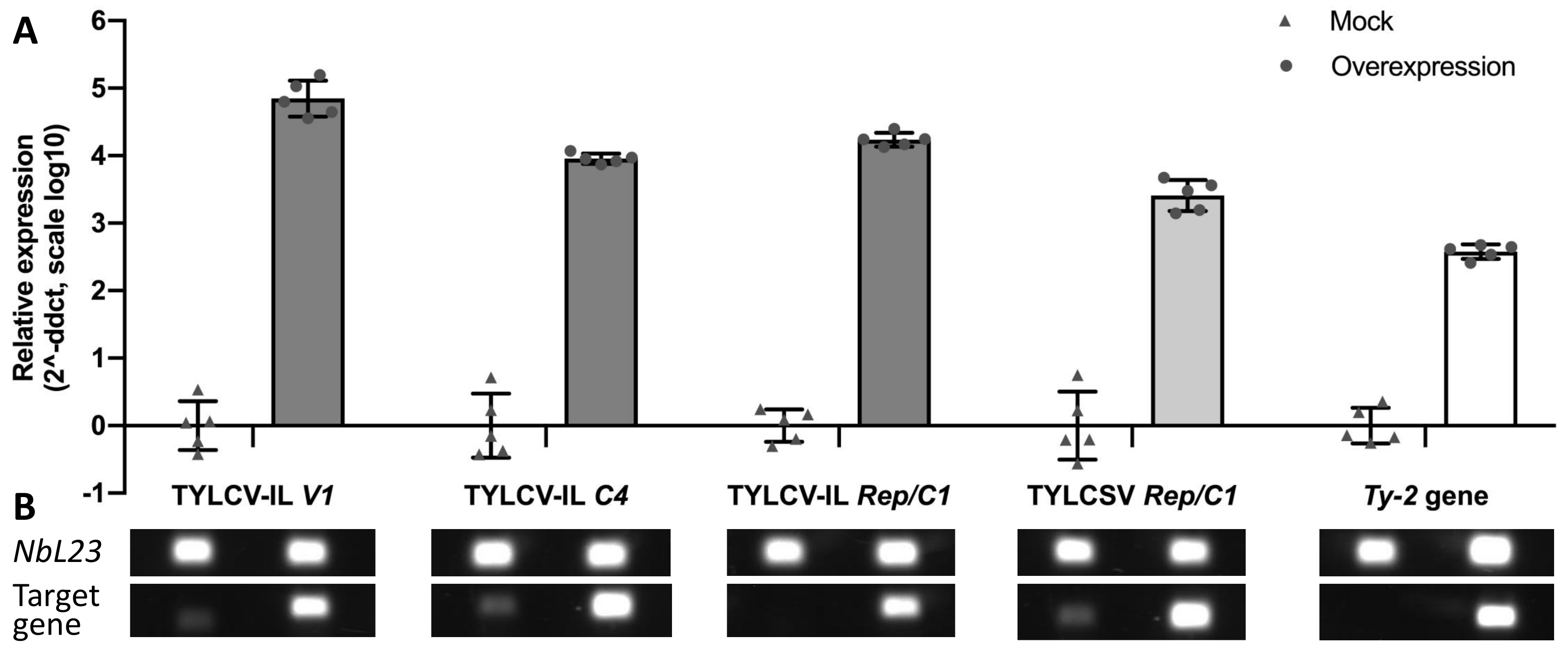

Supplement: Supplementary Figure S2 — Expression of viral genes and Ty-2 gene in mock-treated and infiltrated leaves of Nicotiana benthamiana. (A) RT-qPCR results showing relative expression of target genes using the N. benthamiana gene NbL23 as reference. Values were normalized against mock control and displayed on a logarithmic scale (Log10). The bars show the average value from five biological samples. Error bars indicate the standard deviation. In addition, individual data points are shown. (B) Gel pictures of RT-qPCR products. [file Image_2.tif]

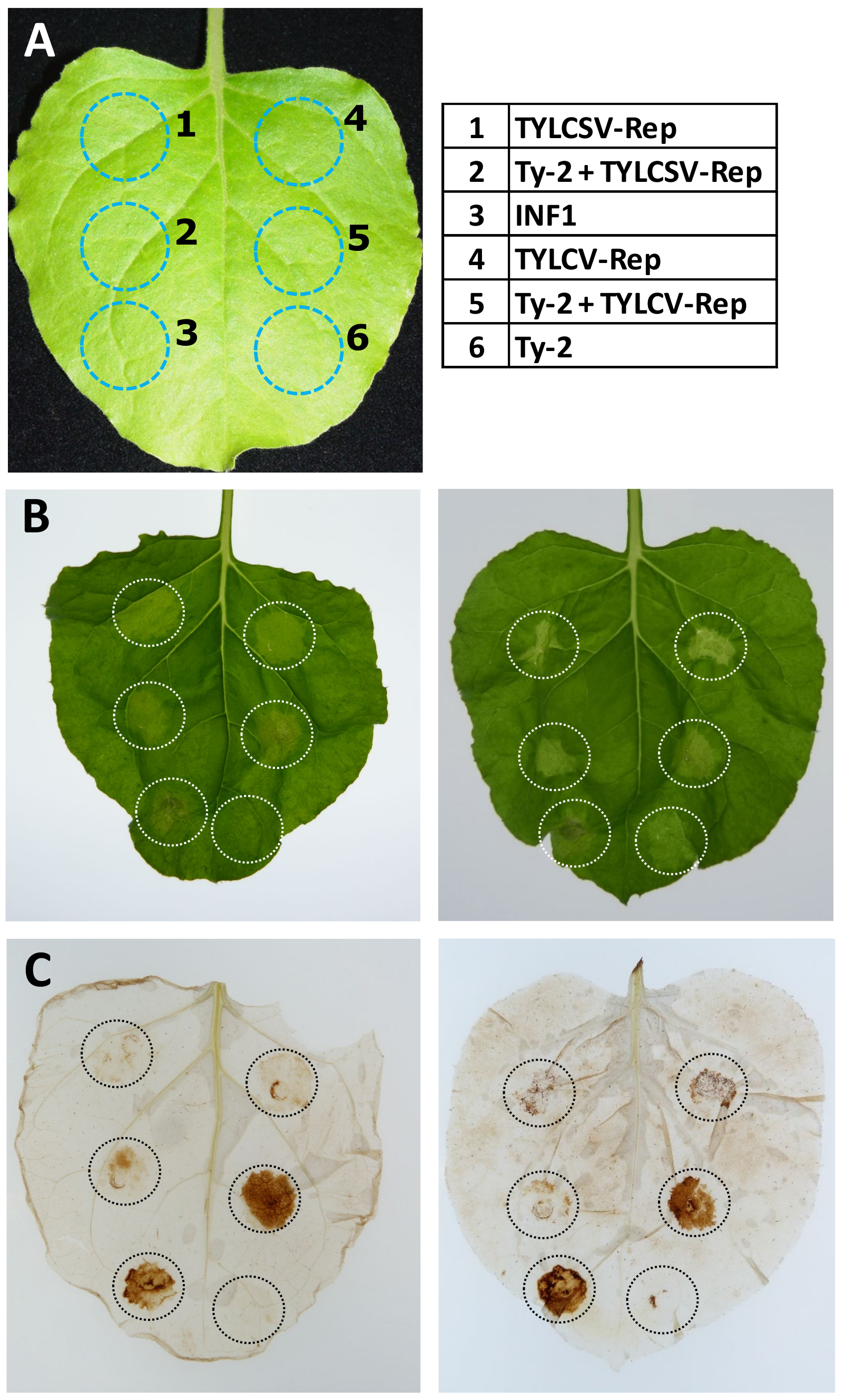

Supplement: Supplementary Figure S3 — Hypersensitive Response after co-infiltration of Ty-2 gene with TYLCV or TYLCSV Rep/C1 gene. (A) Set-up of the co-expression experiment in N. benthamiana. The experiment was repeated three times. In the first experiment ten plants were used, and three leaves per plant were agroinoculated. In the second and third experiment six plants were used. (B) Representative pictures of N. benthamiana leaves three days after infiltration. (C) Results of DAB staining of the same leaves as shown in (B). [file Image_3.tif]
